# Systemic Administration of Erythropoietin Inhibits Retinopathy in RCS Rats

**DOI:** 10.1371/journal.pone.0104759

**Published:** 2014-08-13

**Authors:** Weiyong Shen, Sook H. Chung, Mohammad R. Irhimeh, Shiying Li, So-Ra Lee, Mark C. Gillies

**Affiliations:** 1 Save Sight Institute, the University of Sydney, Sydney, Australia; 2 Southwest Hospital/Southwest Eye Hospital, Third Military Medical University, Chongqing, China; Children's Hospital Boston, United States of America

## Abstract

**Objective:**

Royal College of Surgeons (RCS) rats develop vasculopathy as photoreceptors degenerate. The aim of this study was to examine the effect of erythropoietin (EPO) on retinopathy in RCS rats.

**Methods:**

Fluorescein angiography was used to monitor retinal vascular changes over time. Changes in retinal glia and vasculature were studied by immunostaining. To study the effects of EPO on retinal pathology, EPO (5000 IU/kg) was injected intraperitoneally in 14 week old normal and RCS rats twice a week for 4 weeks. Changes in the retinal vasculature, glia and microglia, photoreceptor apoptosis, differential expression of p75 neurotrophin receptor (p75^NTR^), pro-neurotrophin 3 (pro-NT3), tumour necrosis factor-α (TNFα), pigment epithelium derived factor (PEDF) and vascular endothelial growth factor-A (VEGF-A), the production of CD34^+^ cells and mobilization of CD34^+^/VEGF-R2^+^ cells as well as recruitment of CD34^+^ cells into the retina were examined after EPO treatment.

**Results:**

RCS rats developed progressive capillary dropout and subretinal neovascularization which were accompanied by retinal gliosis. Systemic administration of EPO stabilized the retinal vasculature and inhibited the development of focal vascular lesions. Further studies showed that EPO modulated retinal gliosis, attenuated photoreceptor apoptosis and p75^NTR^ and pro-NT3 upregulation, promoted the infiltration of ramified microglia and stimulated VEGF-A expression but had little effect on TNFα and PEDF expression. EPO stimulated the production of red and white blood cells and CD34^+^ cells along with effective mobilization of CD34^+^/VEGF-R2^+^ cells. Immunofluorescence study demonstrated that EPO enhanced the recruitment of CD34^+^ cells into the retina.

**Conclusions:**

Our results suggest that EPO has therapeutic potentials in treatment of neuronal and vascular pathology in retinal disease. The protective effects of EPO on photoreceptors and the retinal vasculature may involve multiple mechanisms including regulation of retinal glia and microglia, inhibition of p75^NTR^-pro-NT3 signaling together with stimulation of production and mobilization of bone marrow derived cells.

## Introduction

Neuro-vascular degeneration and neovascularization are features of many retinal diseases such as diabetic retinopathy, inherited retinal degenerations and retinopathy of prematurity [1], [2], [3]. The earliest signs of diabetic retinopathy are associated with regional failure of retinal microvascular function, including loss of pericytes and vascular endothelial cells, blood-retinal barrier breakdown, microaneurysms and intraretinal haemorrhages [2], [4]. These vascular abnormalities can lead to macular oedema, retinal ischemia and neovascularization without treatment. Recent studies have reported photoreceptor atrophy in diabetic macular edema, indicating that primary neuronal dysfunction is another important feature of diabetic retinopathy [5], [6]. Retinal degeneration in inherited retinal diseases are usually attributable to specific gene defects in photoreceptors or retinal pigment epithelium cells which lead to photoreceptor dysfunction and death [7]. Inherited retinal degenerations are characterized by progressive night blindness, visual field loss, optic nerve atrophy, retinal vascular degeneration and altered vascular permeability [1], [8]. Vascular degeneration in inherited retinal degenerations is believed to be secondary to diminished metabolic demand in the face of neuronal degeneration. Neovascularization can occur during the later stages of dystrophic, pan-retinal degeneration. Retinopathy of prematurity is another potentially blinding disease affecting premature infants that is characterized by peripheral retinal ischemia and secondary neovascularization [3], [9]. Regardless of its cause, one way to reduce the risk of blindness from retinal neovascularization is to prevent the retinal vasculopathy that precedes and causes it.

The Royal College of Surgeons (RCS) rat develops an inherited retinal phenotype in which shed photoreceptor outer segment debris accumulates in the subretinal space [10]. Positional cloning has identified mutation of c-mer proto-oncogene tyrosine kinase (*MERTK*) in RCS rat and mutations in the human *MERTK* gene are responsible for some cases of autosomal recessive retinitis pigmentosa [10], [11]. In RCS rats, photoreceptor disk shedding commences at postnatal day 12 (P12) and an outer segment debris layer is readily apparent at P20. The time course of photoreceptor degeneration is rapid, beginning around P20, with few photoreceptor nuclei remaining in the outer nuclear layer by P60 [10], [12]. There is evidence that loss of photoreceptors in animal models of retinal degenerations has a profound effect on vascular development of the retina [8]. Recent studies indicate that the progressive loss of photoreceptors in RCS rats is accompanied by capillary dropout and growth of subretinal neovascularization [13], [14].

Erythropoietin (EPO) is an oxygen-regulated hormone produced in the kidney. While the systemic function of EPO is to stimulate erythrocyte formation in response to hypoxia, there is ample evidence that the role of EPO extends beyond erythrogenesis. EPO has been reported to be a powerful cytoprotective factor that protects both neurons and vascular cells from apoptosis and mobilizes bone marrow progenitor cells to the peripheral bloodstream for vascular repair [15], [16], [17], [18], [19], [20], [21], [22]. Intravitreal injection of EPO protects retinal vascular degeneration in the early stages of diabetic retinopathy in streptozotocin-induced diabetic rats [23]. Short-term systemic administration of exogenous EPO in the early stages of retinal ischemia improves retinal neuronal function, prevents retinal capillary dropout and subsequent neovascularization in an animal model of oxygen-induced retinopathy [18], [20]. In order to study further the therapeutic mechanisms underlying EPO treatment for retinal diseases, we examined the effect of EPO on retinal capillary dropout and pathological neovascularization in RCS rats. We paid particular attention to the effect of EPO treatment on retinal glia and microglia, photoreceptor apoptosis, the differential expression of a number of molecules which may affect retinal neurons and blood vessels as well as the production and mobilization of bone marrow-derived progenitor cells following systemic administration of EPO.

## Materials and Methods

### Animals, anaesthesia and pupil dilation

Animal studies were performed in accordance with the Association for Research in Vision and Ophthalmology statement and were approved by The University of Sydney Animal Ethics Committee. Dystrophic RCS rats and non-dystrophic congenic controls were used in this study. The animals were bred in the animal facility of University of Sydney from colonies which were originally provided by Dr. Chooi-May Lai (Lions Eye Institute, the University of Western Australia). For procedures that required general anesthesia, rats were anaesthetised by intraperitoneal injection of ketamine (60 mg/kg) and xylazine (6 mg/kg), and pupils dilated with 2.5% phenylephrine and 1% tropicamide.

### Fundus fluorescein angiography and image analysis

Fundus fluorescein angiography (FFA) was performed to monitor retinal vascular changes in RCS rats. Under general anaesthesia, FFA was conducted after intraperitoneal injection of 0.3∼0.4ml of 10% sodium fluorescein using a modified clinical fundus camera (Topcon TRC-50VT) as described previously [24]. Quantitative analysis of fluorescein leakage on angiograms was performed using the freehand selection tool of the Photoshop software (Photoshop; Adobe Systems) as previously described by Banin et al [25]. Lesions showing leakage ∼2 min after fluorescein injection were specifically selected with the “magic wand” tool in the software (Photoshop; Adobe Systems) and the total area of fluorescein leakage was computed. Measurement of the leaking area was calculated as a percentage of the whole area on angiogram.

### EPO treatment

To examine the effect of EPO treatment on retinal neuronal and vascular pathology, recombinant human EPO (Epoetin alfa, Janssen-Cilag, Australia) was administered by intraperitoneal injection in dystrophic RCS rats and non-dystrophic controls at 14 weeks of age. Each animal was weighed at the day of treatment and then received EPO with a dose of 5000 IU/kg, twice a week for 4 consecutive weeks. This dose has been reported to prevent retinal neuronal apoptosis and vascular dropout in a mouse model of oxygen-induced retinopathy [20].

### Immunofluorescence studies on frozen sections and retinal wholemounts

Eyes were fixed in 4% paraformaldehyde for 1 hr and embedded in optimal cutting temperature compound as described previously [26]. For immunofluorescence staining, frozen sections were blocked with 5% normal goat serum and incubated with primary antibodies against collagen type IV (rabbit polyclonal, 1∶250, AbD serotec, sc-2150-1470),vimentin (mouse monoclonal, conjugated with Cy3, 1∶500, Sigma, Cat# C9080), glial fibrillary acidic protein (GFAP, rabbit polyclonal, 1∶250; Dako #Z0334) and ionized calcium binding adaptor molecule 1 (Iba1, rabbit polyclonal, 1∶500, Wako #019-19741)at +4°C overnight. The bound primary antibodies were detected with a secondary antibody conjugated with Alexa Fluor 488 or 594 (1∶1000; Invitrogen).

For retinal wholemount staining, dissected eye cups were fixed in 4% paraformaldehyde for 1 hr and then placed in PBS at +4°C. The next day, retinas were isolated, permeabilized with 1% Triton-X-100 containing 5% normal goat serum and incubated in a solution containing an antibody to vimentin (vimentin-Cy3, 1∶500, Sigma, C9080) or GFAP (1∶250; Dako) to label glial cells and fluorescence-conjugated *Griffonia simplicifolia* isolectin B4 (IB4, 10 µg/ml; Sigma) for blood vessels. As IB4 also weakly stains microglia in retinal wholemounts, before enucleation some eyes were perfused with fluorescence labelled dextran (FITC-dextran) to clearly reveal the retinal vasculature. In brief, under deep anesthesia, the chest cavity was opened and a cannula was introduced into the aorta to clear blood with saline (500 ml/kg) from the circulation. Retinal perfusion was conducted with FITC-dextran (MW =  2000 kd, 50 mg/kg, Sigma). To examine the recruitment of CD34+ cells into the injured retinal vasculature, retinal wholemounts perfused with FITC-dextran were also stained using an antibody against CD34 (Santa Cruz, sc-7045, 1∶50). Retinal wholemounts were examined by confocal laser scanning microscopy. Quantitative analysis of retinal vasculature and microglial infiltration on retinal wholemounts and frozen sections were performed using computer-based analysis software as previously described [26], [27], [28], [29].

### Terminal deoxynucleotidyl transferase dUTP nick end labeling (TUNEL)

Frozen sections were examined for cell apoptosis using Roche in situ cell death detection kit (Catalog No. 11684795910; Roche Applied Science) according to the manufacturer's instructions followed by propidium iodide nuclear counterstaining (10 µg/ml; Molecular probes) for confocal laser scanning microscopy.

### Western blot

Proteins were extracted from retinas and their concentrations determined by detergent compatible protein assay. Equal amounts of protein were subjected to SDS-polyacrylamide gel electrophoresis then transferred to a high-quality polyvinylidene difluoride membrane. Membranes were probed with primary antibodies to GFAP (goat polyclonal, 1∶500, Abcam, #ab53554), glutamine synthetase (GS, mouse monoclonal, 1∶1000, Millipore #MAB302), p75 neurotrophin receptor (p75^NTR^, rabbit polyclonal, 1;1000, Covance #PRB-602C), precursor form of neurotrophin 3 (pro-NT3, rabbit polyclonal, 1∶500, Alomone laboratory #ANT-012), tumour necrosis factor-α (TNFα, rabbit polyclonal, 1∶2000, Millipore #AB2148P), pigment epithelium derived factor (PEDF, goat polyclonal, R&D Systems # AF1149) and vascular endothelial growth factor –A (VEGF-A, mouse monoclonal, 1∶1000, Abcam #ab1316). After incubation with secondary antibodies conjugated with horseradish peroxidise, protein bands were visualised using the G:Box BioImaging system and quantified using the GeneTools image scanning and analysis package. Protein expression was normalised to α/β tubulin or β-actin which served as loading controls.

### Peripheral blood and bone marrow collection and preparation

Approximate 2∼3 ml of blood was collected into a heparin sodium blood tube from the inferior vena cava of each animal under general anaesthesia. Full blood count was performed with an automated haematology analyser (KX-21N, Sysmex, Japan). To analyse the capacity of EPO treatment to mobilize bone marrow cells to the circulation, red blood cells were lysed using 4 volumes of Fluorescence-Activated Cell Sorting (FACS) lysing solution (BD Biosciences, catalogue#349202) and washed twice with 10 volumes of Magnetic Affinity Cell Sorting (MACS) buffer (Miltenyi Biotech, catalogue#130-091-221). Cells were resuspended in 2 ml of MACS buffer and the total number of cells and their viability were determined by automated cell counter (TC10, BioRad, Australia) and trypan blue exclusion assay. In addition, bone marrow was collected following peripheral blood collection. In brief, bone marrow was flushed from femurs and tibias using MACS buffer. Cells were centrifuged, resuspended in MACS buffer and then filtered through a 40 µm mesh (BD Biosciences). After lysing red blood cells, the total number of bone marrow cells and their viability were determined as described previously [19], [30].

### Flow cytometry

Flow cytometric acquisition was performed on a FACSCanto II flow cytometer analyser using FACSDiva software (both from BD Biosciences) as described previously [19], [30]. In brief, cells were Fc-blocked (BD Biosciences, catalogue#550271) with 1 µg/million cells for 15 minutes on ice, and then stained with a designated antibody panel containing CD 45 PE.Cy5 (BD Biosciences, #559135), CD31 FITC (AbD Serotec, #MCA1334F), CD 146 PE (R&D Systems, #FAB3250P) CD90.1 APC (Miltenyi Biotech, #130-094-525) and CD34 PerCp.Cy5.5 (Santa Cruz, #sc-7324). Cells were first gated on forward scatter (FSC) versus side scatter (SSC) to collect only small lymphocytes followed by a discrimination blot (FSC-A versus FSC-H) for singlet population. The gating strategy excluded CD45^+^ cells (a leukocyte common antigen), then cells were gated for analysis of the percentages of CD31^+^/146^+^/90^+^ that were further analyzed for CD31^+^/146^+^/90^+^/CD34^+^ and CD31^+^/146^+^/90^+^/CD34^−^ cells. Furthermore, cells showing CD31^+^/146^+^/90^+^/CD34^+^ were further analyzed for the populations of VEGF-R2^+^ and VEGF-R2^−^ cells using an antibody against Flk-1/VEGF-R2 (Santa Cruz, #sc-6251) in combination with a corresponding secondary antibody conjugated with FITC (Santa Cruz, #sc-2010). Negative control tubes were prepared identically using isotype control antibodies to define “positive populations”. Flow cytometry absolute count beads standard (580, Bangs Laboratories) was used as an internal counting standard for cell enumeration. Beads were mixed with samples at a concentration of 10^5^ beads per sample and gated on FFS-A vs SSC-A, FFS-A vs FFS-H and then followed by channels 530 vs 585.

### Statistic analysis

Results are expressed as mean ± SEM. Data were analysed using unpaired Student t-test. A p value <0.05 was regarded as statistically significant.

## Results

### RCS rats developed progressive vascular leakage during photoreceptor degeneration

**Figure 1:**
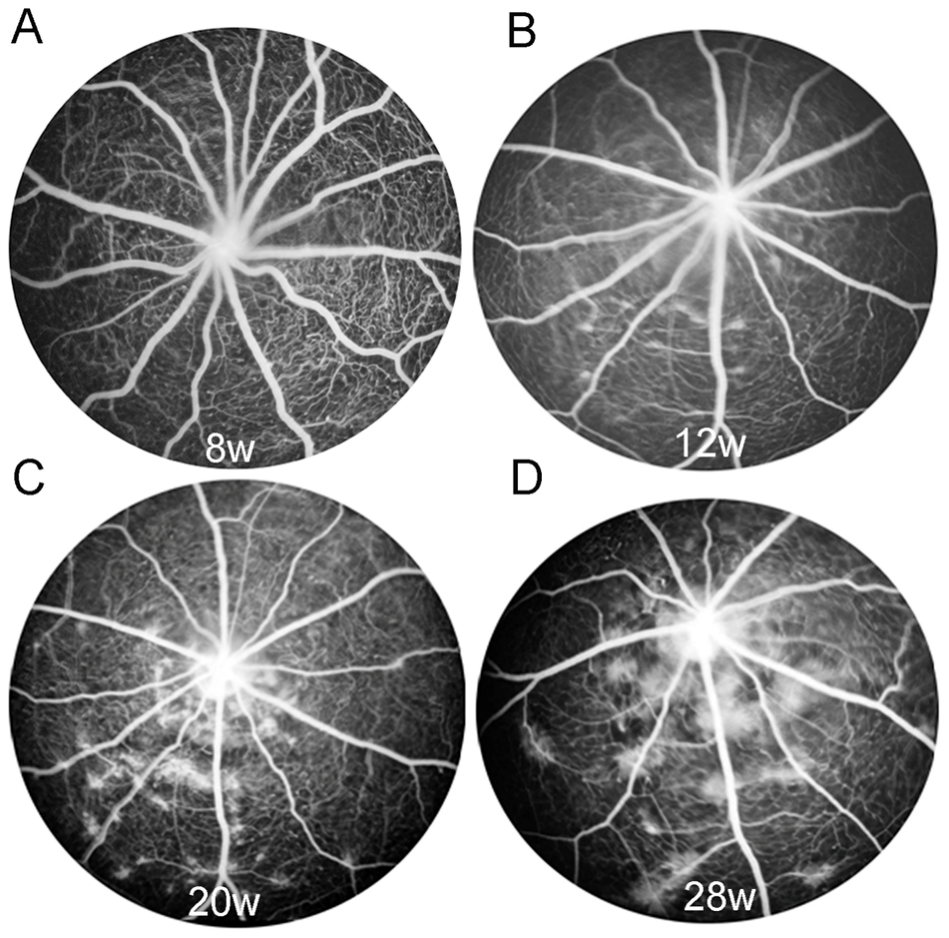
RCS rats develop progressive vascular leakage over the course of retinal degeneration. Fundus fluorescein angiography was performed to monitor changes in the retinal vasculature over time. RCS rats showed relatively normal retinal vasculature at 8 weeks (w) of age (A) but developed vascular leakage from 12 weeks of age (B). The vascular lesions were predominantly confined to regions surrounding the optic disc and became more severe with time.

We performed FFA to monitor changes in the retinal vasculature over the course of photoreceptor degeneration in RCS rats (Figure 1A). FFA showed relatively normal retinal vasculature at 8 weeks of age (Figure 1A). However, RCS rats developed vascular leakage from 12 weeks of age (Figure 1B). The vascular lesions were predominantly confined to regions surrounding the optic disc and progressed in severity with time (Figure 1, C and D).

### The development of vasculopathy were accompanied by retinal gliosis

**Figure 2:**
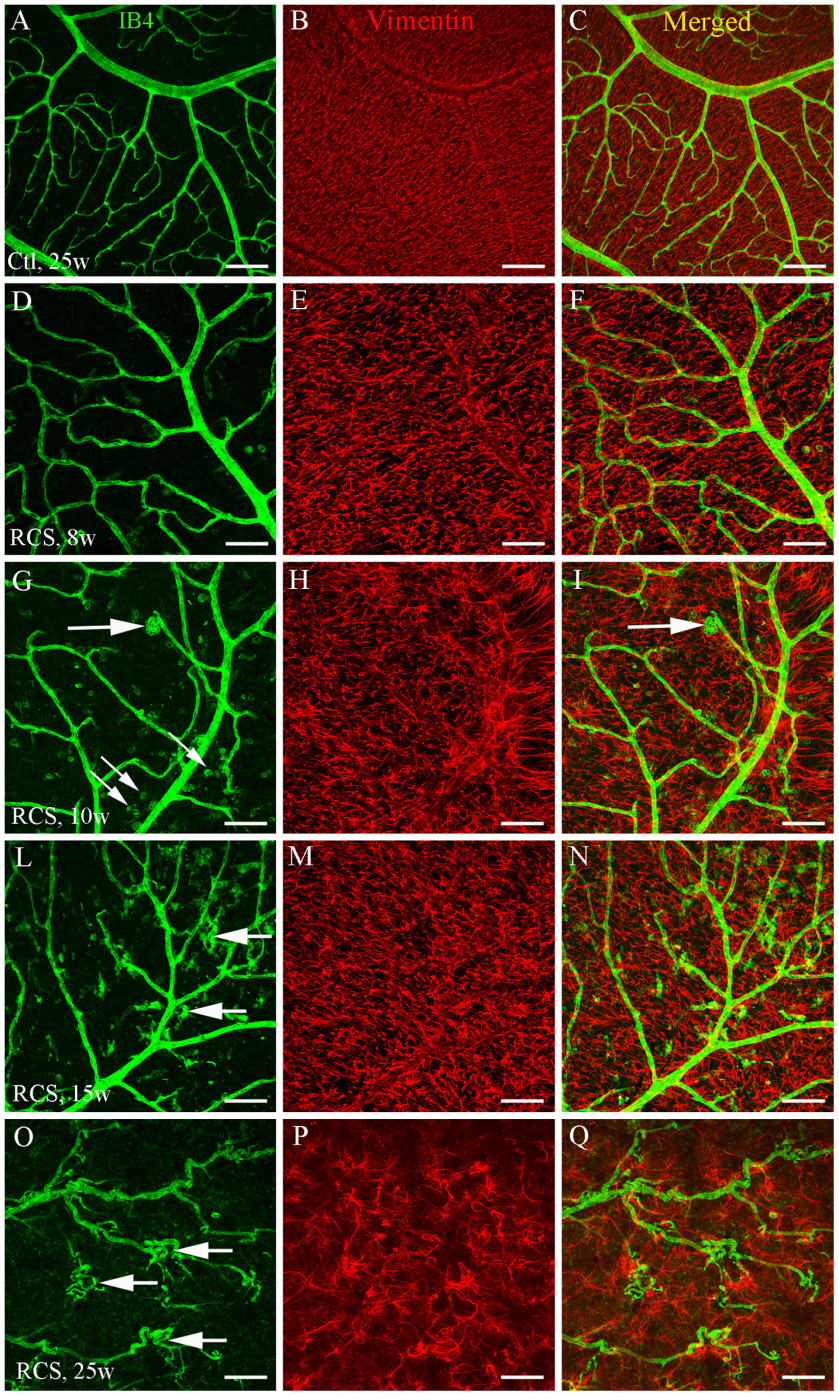
Retinal vascular abnormalities are accompanied by reactive gliosis. Retinal wholemount staining was performed using fluorescence-conjugated *Griffonia simplicifolia* isolectin B4 to label blood vessels (A, D, G, L, O; green) in combination with an antibody against vimentin for glial cells (B, E, H, M, P; red) in retinas isolated from non-dystrophic (A–C) and RCS (D–Q) rats at various ages. (C, F, I, N, Q) merged images. (A–C) the non-dystrophic retina showed smooth, well-defined retinal vessels and glial cells. (D–F) RCS rats at 8 weeks (w) of age showed relatively normal retinal vasculature but with slight dilation of retinal capillaries and activation of retinal glia. (G–Q) Capillary dropout, intraretinal neovascularization (large arrows in G, L, O) and retinal gliosis (H, M, P) progressed with time in RCS rats. RCS rats at 25 w of age developed severe capillary dropout, neovascularization and vascular telangiectasis (O–Q). The small arrows in (G) indicate the infiltration of macrophage-like cells. Scale bars: 100 µm.

Double label immunostaining was performed on retinal wholemounts to map vascular changes with reactive gliosis during retinal degeneration. Non-dystrophic rats demonstrated a smooth and well-defined vascular network which was accompanied by a regular and smooth arrangement of glial cells in the retina (Figure 2, A–C). RCS rats at 8 weeks of age showed mild capillary dropout, dilation of surviving retinal capillaries and activation of retinal glia (Figure 2, D–F). RCS rats developed obvious capillary dropout, neovascularization and strong retinal gliosis at 10 and 15 weeks of age (Figure 2, G–N). After 25 weeks of age, severe capillary dropout, neovascularization and vascular telangiectasis were noted (Figure 2O). Compared with 10 and 15 weeks of age, RCS rats at 25 weeks of age appeared to have lost a large number of glial cells but the surviving glial cells showed prominent features of activation which was evidenced by further enlargement of their cell processes (Figure 2, P and Q).

**Figure 3:**
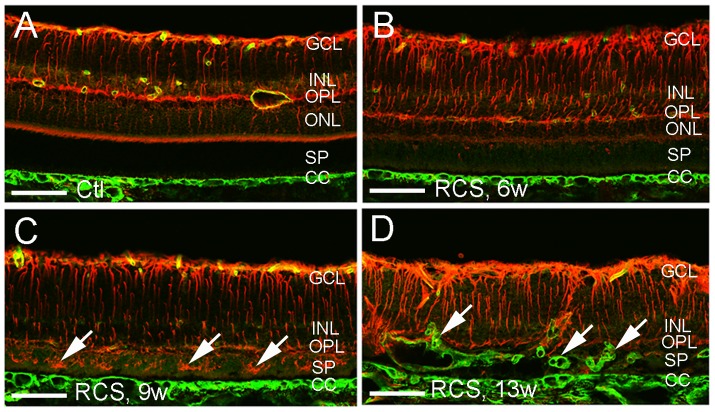
Retinal gliosis and subretinal neovascularization in RCS rats. Double label immunostaining was performed using antibodies against vimentin (red) and collagen type IV (green) on frozen sections. (A) Non-dystrophic rat at 21 weeks (w) of age showed a normal distribution of retinal vessels confined to the inner retina and absence of glial cells in the subretinal space. (B, C) RCS rats at 6 w (B) and 9 w (C) of age showed activation of Muller cells but without subretinal neovascularization. Muller cell invasion into the subretinal space was more obvious at 9 w compared with 6 w of age (arrows in C). (D) Subretinal neovascularization (arrows) in RCS rats at 13 w of age. GCL = ganglion cell layer, INL = inner nuclear layer, OPL = outer plexiform layer, ONL = outer nuclear layer, SP = subretinal space, CC = choroidal capillaries. Scale bars: 50 µm.

We performed double label immunofluorescence to study the development of Muller cell gliosis and subretinal neovascularization during photoreceptor degeneration (Figure 3). Blood vessels of the normal retina were confined to the inner retina and Muller cell processes were not found in the subretinal space (Figure 3A). By 6 to 9 weeks of age, RCS rats had developed severe photoreceptor degeneration which was accompanied by widespread activation of Muller cells across the retina, with progressive invasion of Muller cell processes into the subretinal space without subretinal neovascularization (Figure 3, B and C). By 13 weeks of age, severe loss of photoreceptors was accompanied by pronounced subretinal neovascularization and remarked Muller cell gliosis (Figure 3D).

### Systemic administration of EPO prevented retinal capillary dropout

**Figure 4:**
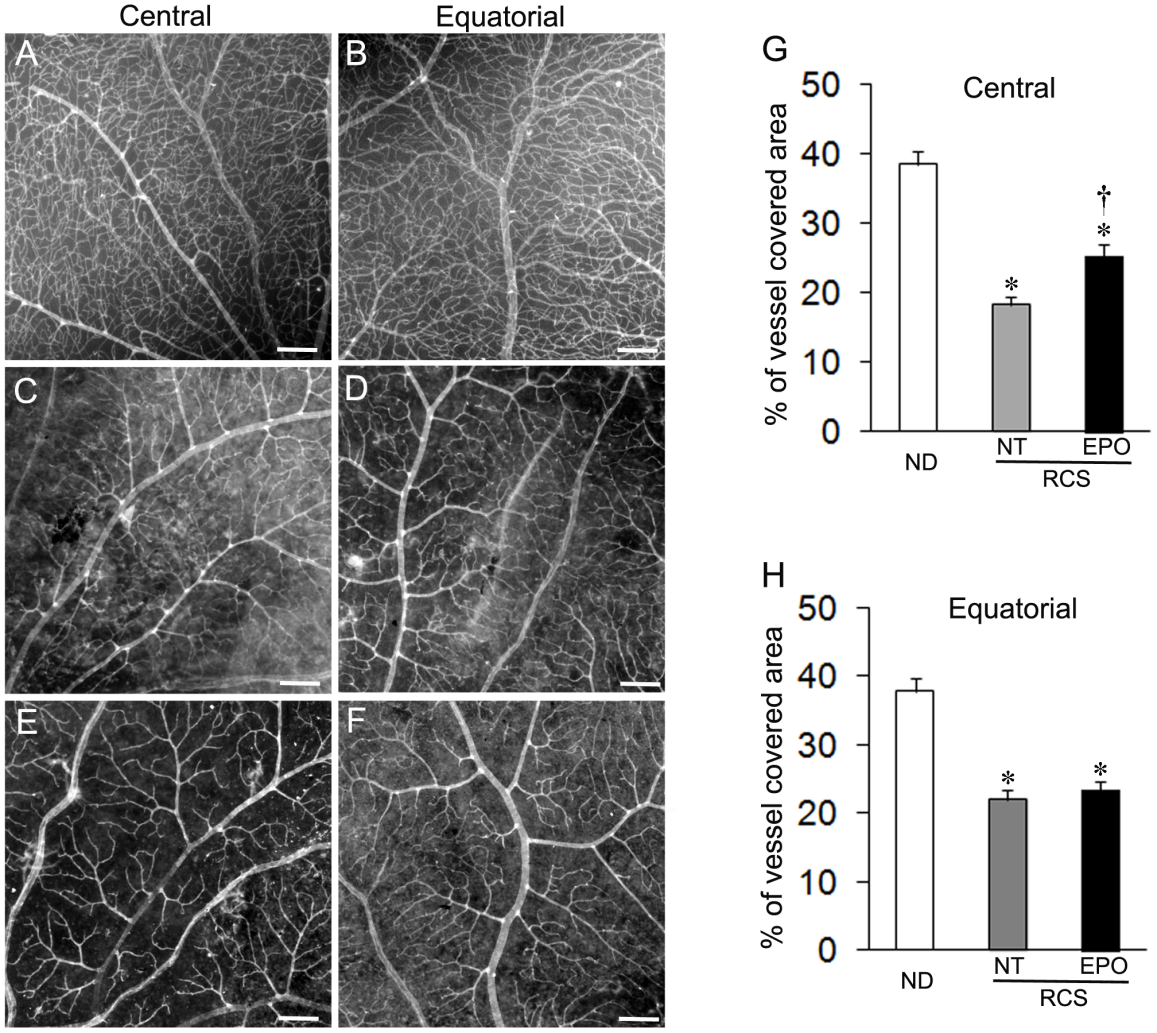
EPO treatment prevents retinal capillary dropout. (A–F) Representative images taken from the central (A, C, E) and equatorial (B, D, F) regions of the retina after perfusion with fluorescence labelled dextran (FITC-dextran). (A, B) Non-dystrophic (ND) rat. (C–D) RCS rats without treatment. (E–F) RCS rats treated with EPO. (G, H) Quantitative analysis of retinal vasculature revealed by FITC-dextran perfusion [27]. ^*^P<0.01, ND vs RCS rats; ^†^P<0.05, RCS rats treated with EPO vs RCS rats not-treated (NT), respectively; n = 6∼8/group. Scale bars: 200 µm.

We next examined changes in retinal vascular density to determine whether EPO treatment prevented retinal capillary dropout (Figure 4). Normal retinas had very dense retinal vascular networks in both central and equatorial regions (Figure 4, A and B). In RCS rats without treatment, however, capillary dropout occurred in both regions (Figure 4, C and D). Systemic administration of EPO prevented capillary dropout in the central region but appeared to be less effective in the equatorial region (Figure 4, E and F). Quantitative analysis of retinal vasculature showed that the vascular density in the central area was significantly higher in EPO treated group compared animals without treatment (Figure 4G). There was no statistically significant difference in the density of the equatorial vascular networks between EPO treated and untreated groups (Figure 4H).

### EPO treatment prevented the development of focal vascular lesions

**Figure 5:**
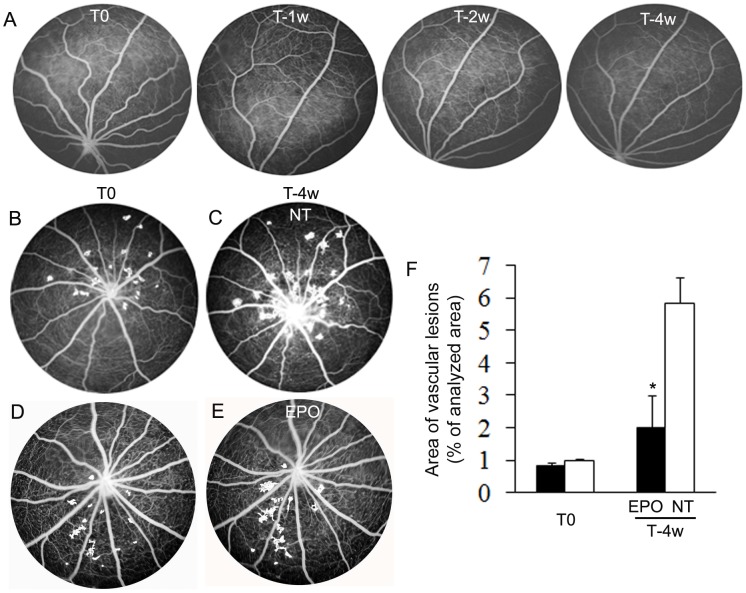
Systemic administration of EPO prevents intraretinal neovascularization. EPO was injected at 14 weeks of age (T0), twice a week for 4 consecutive weeks. (A) Fundus fluorescein angiography (FFA) in a non-dystrophic rat before (T0), 1 week (T-1w), 2 weeks (T-2w) and 4 weeks (T-4w) after EPO treatment. (B–E) FFA in RCS rats at 14 weeks of age (T0, B and D) and 4 weeks (T-4w, C and E) after EPO treatment. (F) Quantitative analysis of vascular lesions [25] as demonstrated in (B–E) in RCS rats before and 4 weeks after EPO treatment. ^*^P<0.01, RCS rats treated with EPO vs RCS rats not treated (NT); n = 6/group.

We next examined whether long-term systemic administration of recombinant EPO inhibited the development of retinal neovascularization (Figure 5). We documented the development of focal vascular lesions before and after EPO treatment in real time using FFA. Four weeks of EPO treatment did not affect the retinal vasculature of non-dystrophic rats (Figure 5A). Consistent with our previous observations, RCS rats without EPO treatment developed foci of intraretinal neovascularization at 14 weeks of age which progressed with time (Figure 5, B and C). Four weeks of EPO treatment prevented the progressive development of these lesions (Figure 5, D and E). Quantitative analysis of FFA images showed that the area of vascular leakage from focal lesions was significantly smaller in EPO treated RCS rats compared with untreated RCS rats (Figure 5F).

### EPO treatment inhibited retinal gliosis

**Figure 6:**
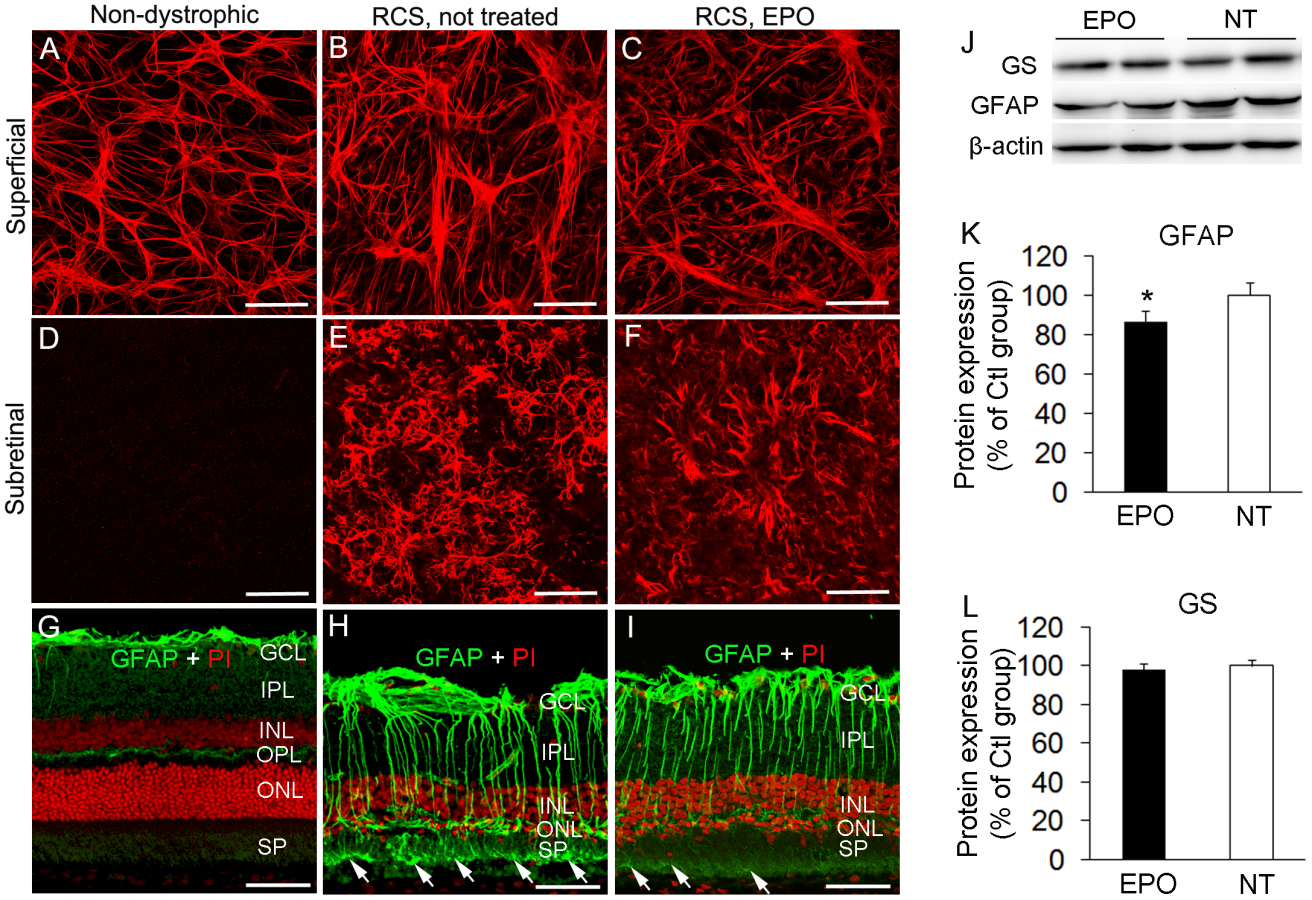
EPO treatment modulates retinal gliosis. (A–F) Retinal wholemount immunostaining for glial fibrillary acidic protein (GFAP) in retinas isolated from non-dystrophic (A, D) and RCS (B, C, E, F) rats with (C, F) and without (A, B, D, E) EPO treatment. (G–I) Immunofluorescence for GFAP (green) and propidium iodide (PI, red) nuclear counterstaining in non-dystrophic (G) and RCS rats with (I) and without (H) EPO treatment. Arrows in (H and I) point to Muller cell processes protruding into the subretinal space. Scale bars: A–F, 100 µm. G–I, 50 µm. GCL = ganglion cell layer, IPL = inner plexiform layer, INL = inner nuclear layer, OPL = outer plexiform layer, ONL = outer nuclear layer, SP = subretinal space. (J) Western blots using antibodies to detect GFAP and glutamine synthetase (GS). (K, L) Quantitative analysis of protein band densitometry. ^*^P<0.05, EPO treated vs not-treated (NT) RCS rats; n = 4/group.

Since long-term gliosis may exert detrimental effects on retinal neurons and blood vessels [24], [31], we performed immunofluorescence and Western blots to examine whether EPO treatment inhibited retinal gliosis (Figure 6). Immunofluorescent studies on retinal wholemounts showed that GFAP immunoreactivity was restricted to astrocytes in the superficial retina of non-dystrophic rats (Figure 6, A and D). Strong GFAP immunoreactivity was observed in superficial retina and the subretinal space of 18 week old RCS rats, indicating marked activation of astrocytes and Muller cells (Figure 6, B and E). Systemic EPO treatment reduced GFAP immunoreactivity at both levels (Figure 6, C and F). Consistent with our findings from retinal wholemounts, immunostaining of frozen sections showed GFAP expression was confined to the superficial retina and outer plexiform layer in non-dystrophic rats (Figure 6G) but was strongly upregulated across the entire neuroretina of dystrophic RCS rats in which Muller cell processes frequently invaded into the subretinal space (Figure 6H, arrows). EPO treatment reduced the extent of Muller cell process invasion into the subretinal space (Figure 6I). Western blots showed that EPO significantly reduced the level of GFAP expression whereas GS expression was not affected by EPO treatment (Figure 6, J–L).

### EPO treatment inhibited photoreceptor apoptosis, tended to increase ramified microglial infiltration and regulated differential expression of p75^NTR^, pro-NT3 and VEGF-A

**Figure 7:**
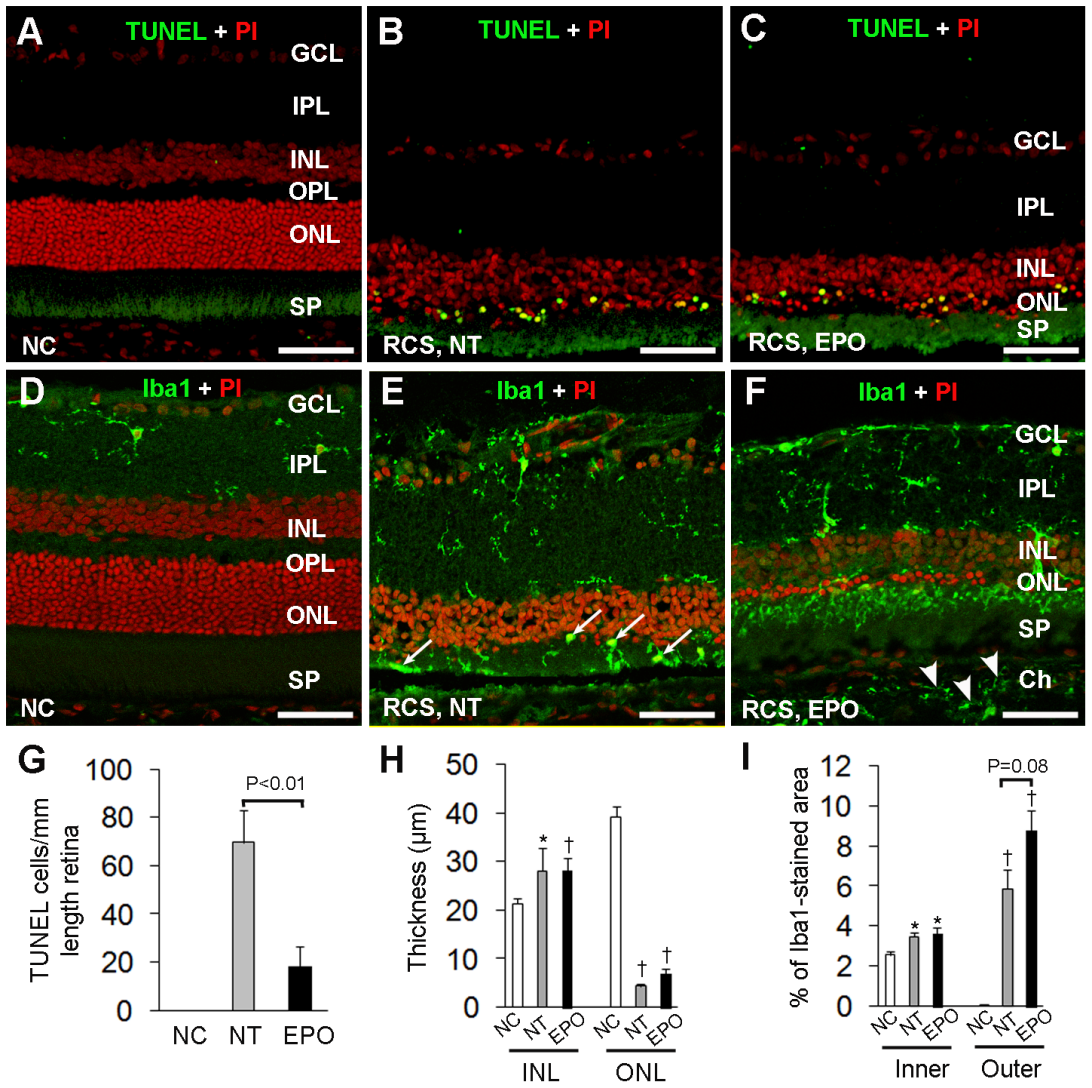
EPO protects photoreceptors and increases ramified microglia infiltration in the subretinal space. (A–C) TUNEL staining (green) in retinas of non-dystrophic (A) and RCS rats with (C) and without (B) EPO treatment. (D–F) Immunofluorescence for ionized calcium binding adaptor molecule 1 (Iba1, green) and propidium iodide (PI, red) nuclear counterstaining on frozen sections produced from the above animals. Arrows in (E) point to microglia with enlarged cell somas and retracted cell processes in the subretinal space. Arrowheads in (F) point to increased microglia in the choroid. Note: more ramified morphology of microglia in the subretinal space in (F) compared with the microglia in (E). Scale bars: A–F, 50 µm. NC = non-dystrophic controls. NT = not treated. GCL = ganglion cell layer, IPL = inner plexiform layer, INL = inner nuclear layer, OPL = outer plexiform layer, ONL = outer nuclear layer, SP = subretinal space, Ch = choroid. (G–I) Quantitative analysis of TUNEL positive photoreceptors (G), the thickness of INL and ONL (H) and microglial infiltration (I). ^*^P<0.05 and ^†^P<0.01, vs non-dystrophic control (NC) group; n = 6/group.

We next examined the effects of EPO treatment on photoreceptor apoptosis and microglial infiltration (Figure 7). TUNEL^+^ cells were not detected in the normal retina (Figure 7A). By 18 weeks of age, untreated RCS rats had only one or two rows of photoreceptor nuclei and most of the surviving photoreceptors were TUNEL^+^ (Figure 7B). EPO treatment significantly reduced the number of TUNEL^+^ photoreceptors (Figure 7, C and G) but this protective effect did not increase the thickness of outer nuclear layer (Figure 7H) because EPO treatment was conducted at 14 weeks of age by which time the photoreceptor layer had almost completely degenerated [10], [12].

We conducted immunofluorescence studies to evaluate the effect of EPO treatment on microglia in degenerating retinas. In non-dystrophic rats, ramified microglia were confined to the superficial retina and inner plexiform layer but absent in the outer retina (Figure 7D). In un-treated RCS rats, marked activation of microglia was observed across the retina (Figure 7E). Most microglia that were infiltrating into the subretinal space had enlarged somas and retracted cell processes (Figure 7E, arrows). Quantitative analysis of Iba1-labelled microglia indicated that EPO treatment tended to increase Iba1-labeled microglia in the outer retina (P = 0.08, Figure 7I). In EPO-treated eyes, most microglia in the subretinal space showed more ramified morphology with smaller somas (Figure 7F) compared with un-treated RCS rats (Figure 7E). EPO treatment also appeared to result in increased infiltration of ramified microglia into the choroid (Figure 7F, arrowheads).

**Figure 8:**
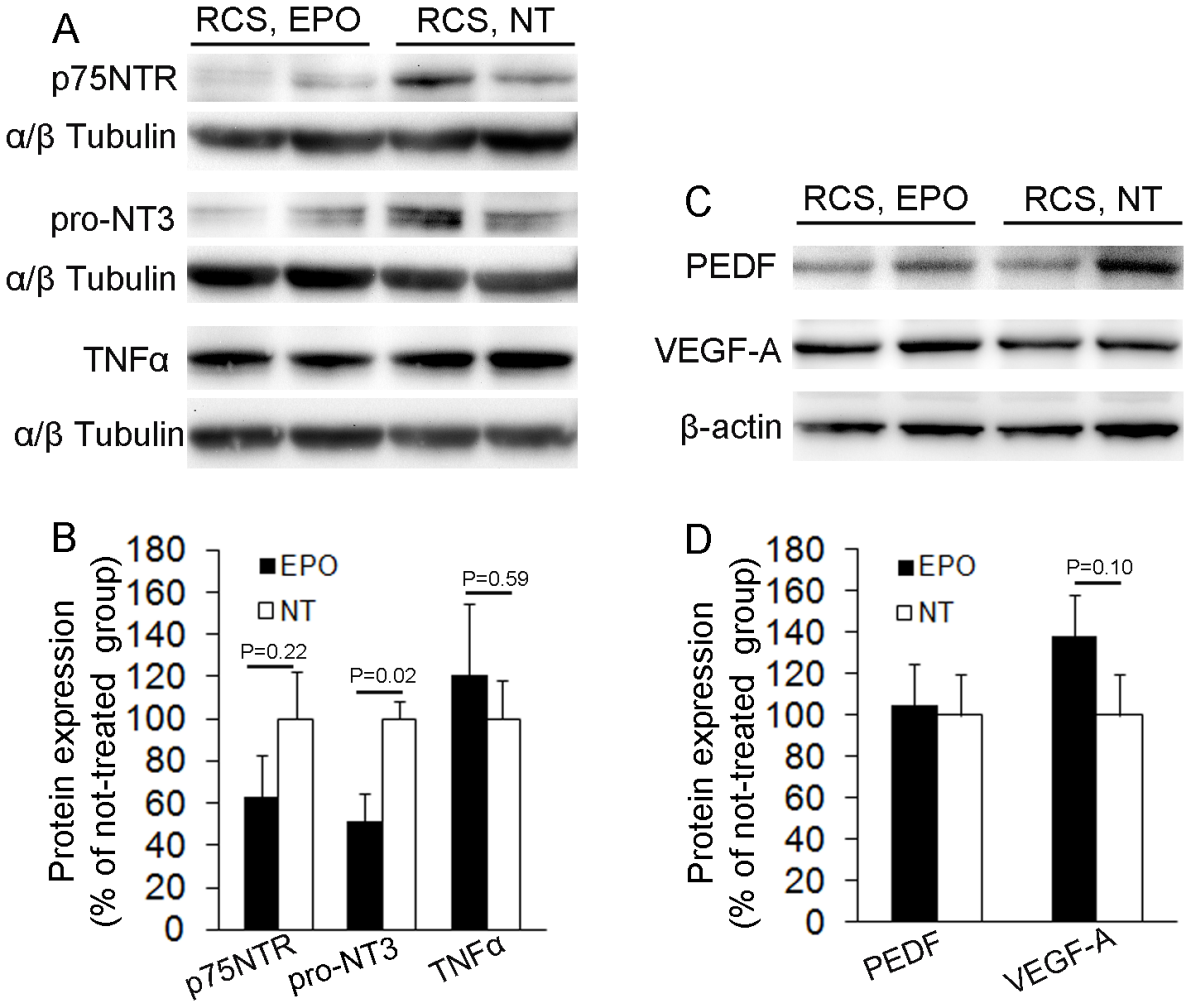
The effects of EPO on differential expression of p75^NTR^, pro-NT3 and VEGF-A. (A and C) Western blots using antibodies to detect p75 neutrotropin receptor (p75^NTR^), the precursor form of neurotrophin 3 (pro-NT3), tumor necrosis factor alpha (TNFα), pigment epithelium derived factor (PEDF) and vascular endothelial growth factor A (VEGF-A). (B, D) Quantitative analysis of protein band densitometry, n = 4/group.

Since activated Muller cells and microglia can influence neurons and blood vessels through upregulation of p75^NTR^ [29], [32], pro-neurotrophins [29], [33], cytokines [28], [33] and angiogenic factors [13], [34], we performed Western blots to examine the effects of EPO treatment on p75^NTR^, pro-NT3, TNFα, VEGF-A and PEDF expression (Figure 8). We found that EPO tended to reduce p75^NTR^ (P = 0.22) and significantly decreased pro-NT3 (P = 0.02) expression but did not affect TNFα expression (Figure 8, A and B). Western blots for PEDF and VEGF-A demonstrated that EPO increased VEGF-A expression by 38% (P = 0.10) but did not affect PEDF expression compared with RCS rats not receiving treatment (Figure 8, C and D).

### EPO stimulated the production of blood cells including CD34^+^ cells and effectively mobilized CD34^+^/VEGF-R2^+^ cells from bone marrow to the circulation

**Figure 9:**
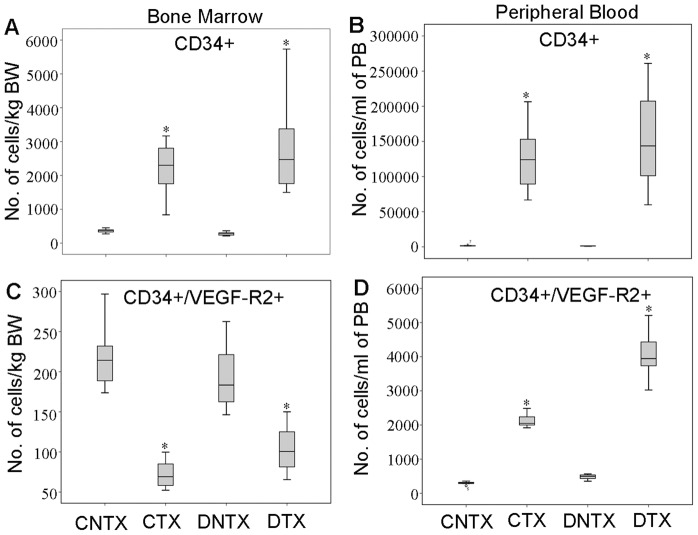
EPO treatment enhances the production of CD34^+^ cells and mobilizes CD34^+^/VEGF-R2^+^ cells from bone marrow to peripheral blood. (A–D) Flow cytometric analysis of the populations of CD34^+^ and CD34^+^/VEGF-R2^+^ cells isolated from bone marrow (BM) and peripheral blood (PB). CNTX = non-dystrophic control rats without EPO treatment, CTX = non-dystrophic control rats receiving EPO treatment, DNTX = RCS rats without EPO treatment, DTX = RCS rats receiving EPO treatment. Box plots display the 25^th^ percentile, median (50^th^ percentile), 75^th^ percentile as well as the minimum and maximum number of cells analyzed. *P<0.01, EPO treated group vs not treated group; n = 7∼10/group.

We analyzed a number of parameters to examine the effect of systemic EPO treatment on peripheral blood cell production (Table 1). EPO treatment significantly increased the numbers of red and white blood cells in both normal and RCS dystrophic rats, leading to increased levels of hamatocrit, haemoglobin, mean corpuscular volume and red cell distribution width (Table 1). After isolation of cells from bone marrow and peripheral blood, we analyzed changes in the number of CD34^+^ and CD34^+^/VEGF-R2^+^ cells using flow cytometry to determine the effectiveness of EPO in stimulating the production and mobilization of bone marrow progenitor cells (Figure 7). We found that EPO treatment significantly increased the number of CD34^+^ cells in the bone marrow and peripheral blood in normal and RCS dystrophic rats (Figure 9, A and B). Whereas the number of CD34^+^/VEGF-R2^+^ cells was significantly reduced in the bone marrow (Figure 9C), the number of CD34^+^/VEGF-R2^+^ cells was significantly increased in the peripheral blood after EPO treatment (Figure 9D).

**Table 1:** Peripheral blood analysis after EPO treatment in normal and RCS dystrophic rats.

Item	Control	Control-EPO	RCS	RCS-EPO
RBC (x 10^12^/L)	6.27±0.18	8.14±0.24^**^	7.82±0.60	9.20±0.93^*^
Hct (%)	0.35±0.01	0.45±0.01^**^	0.44±0.03	0.56±0.06^**^
Hb (g/L)	116±2.45	151±4.35^**^	144±10.23	181±20.04^**^
MCV (fL)	56±0.52	60±2.31^**^	56±1.13	61±2.80^**^
RDW (fL)	27±0.38	41±5.15^**^	29±0.53	44±6.29^**^
WCC (x 10^9^/L)	8.42±1.73	10.90±2.20^**^	10.40±3.09	17.60±3.89^**^
Lym (x10^9^/L)	7.30±1.74	8.88±0.82^*^	9.02±2.03	10.90±1.78

P values were calculated by comparing data from non-dystrophic rats without treatment vs those treated with EPO and RCS rats without treatment vs RCS rats treated with EPO, respectively. ^*^P<0.05 and ^**^P<0.001; Student's t-test; n = 7∼10/group. RBC = red blood cells, Hct = hematocrit, Hb = Hemoglobin, MCV = mean corpuscular volume, RDW = Red cell distribution width, WCC = white cell count, Lym = lymphocytes.

### EPO treatment resulted in increased recruitment of CD34^+^ cells into the retina

**Figure 10:**
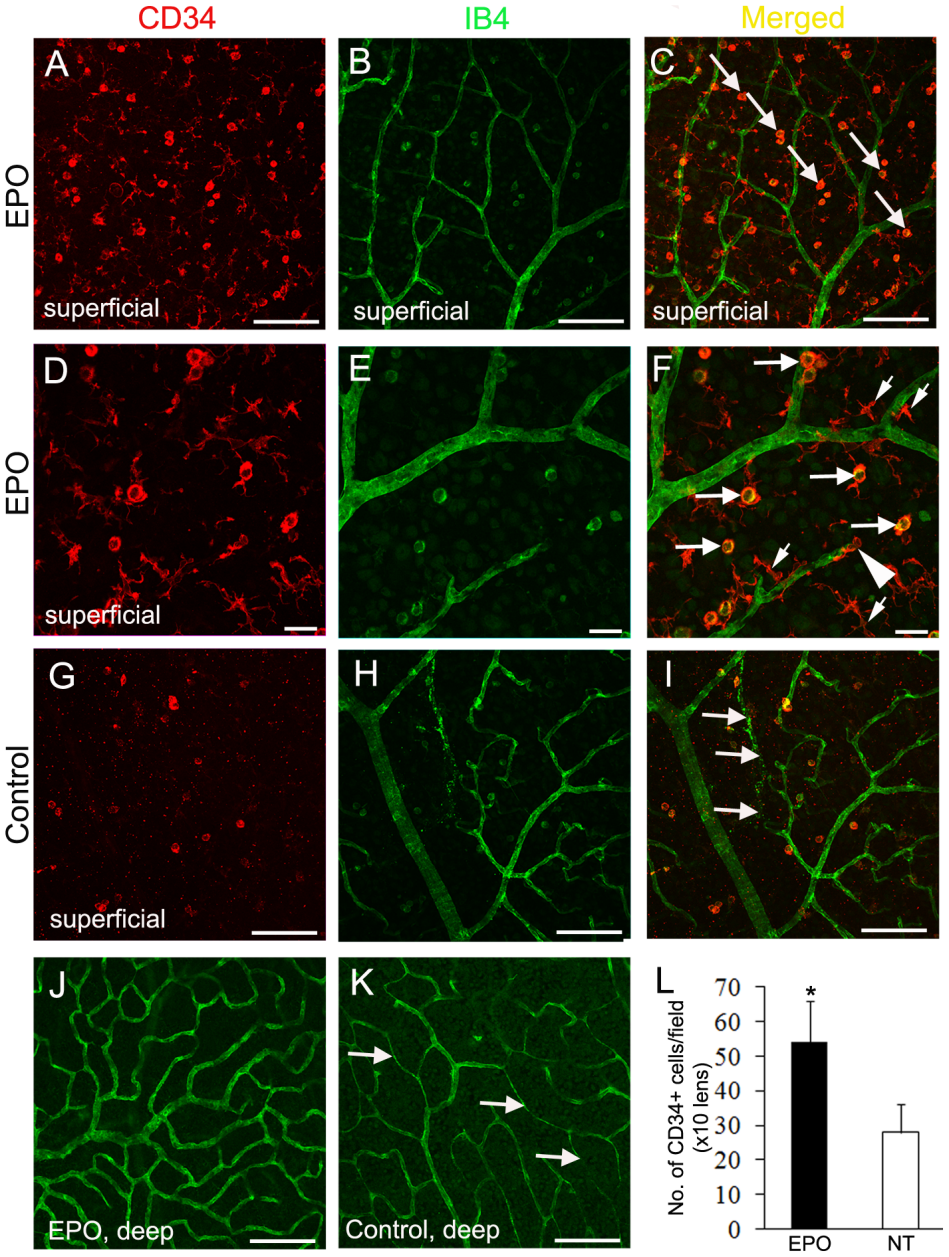
EPO treatment increases the number of CD34^+^ cells in the retina. (A–K) Immunostaining for CD34^+^ cells (red) on retinal wholemounts stained with isolectin B4 (IB4, green). (A–F, J) RCS rats treated with EPO. (G–I, K) RCS rats without EPO treatment. In the EPO treated retina, a large number of CD34^+^ cells with monocyte-like (larger arrows in C and F) and microglia-like (small arrows in F) morphology were detected in the superficial (A–F) rather than the deep (J) vascular plexuses. The arrowhead in (F) points to a CD34^+^ cell wrapping an injured vessel. Arrows in (I) point to degenerating (D) and severely narrowed (K) retinal capillaries in rats without EPO treatment. NT = age-matched RCS rats not treated with EPO. (L) Quantitative analysis of CD34^+^ cells on retinal wholemounts. ^*^P<0.05, RCS rats treated with EPO vs RCS rats not treated group; n = 6/group. Scale bars: 100 µm in (A–C, G–K); 25 µm in (D–F).

As systemic administration of EPO was effective in mobilizing bone marrow cells to the circulation, we reasoned that EPO treatment would increase recruitment of bone marrow derived cells into the retina. Double label staining for CD34 and IB4 on retinal wholemounts showed that EPO treatment markedly increased the number of CD34^+^ cells in the retina, where they were predominantly distributed in the superficial (Figure 10, A–C and G–I) rather than the deep vascular plexuses (Figure 10, J and K). CD34^+^ cells in EPO treated retinas demonstrated monocyte-like and microglia-like morphology (Figure 10, C and F). Most CD34^+^ cells seemed to be scattered randomly in the superficial retina although we occasionally found CD34^+^ cells wrapping injured blood vessels (Figure 10F, arrowhead). The deep vascular plexus appeared to be well preserved in EPO-treated RCS rats (Figure 10J). The retinas of untreated RCS rats showed fewer CD34^+^ cells compared with the EPO-treated group, with the majority of cells showing monocyte-like morphology (Figure 10G). Degenerating and narrowed retinal capillaries were frequently observed in the superficial (Figure 10I) and deep (Figure 10K) vascular plexuses in untreated RCS rats. Quantitative image analysis showed that EPO treatment significantly increased the recruitment of CD34^+^ cells into the retina (Figure 10L).

## Discussion

We describe here the protective effect of systemic EPO treatment on vasculopathy that develops as photoreceptors degenerate in RCS rats. We first confirmed that RCS rats developed progressive retinal capillary dropout, reactive gliosis and subretinal neovascularization. We then found that long term systemic EPO treatment prevented capillary dropout and progression of focal vascular lesions. Further studies indicated that EPO treatment modulated retinal gliosis and ramified microglial infiltration, protected photoreceptors from apoptosis and influenced differential expression of p75^NTR^, pro-NT3 and VEGF-A. EPO stimulated the production of CD34^+^ cells and effectively mobilized CD34^+^/VEGF-R2^+^ cells. Immunofluorescence studies showed that EPO treatment increased the recruitment of CD34^+^ cells to the retina. Taken together, our results suggest that EPO has therapeutic potentials in treatment of neuronal and vascular pathology in retinal disease.

There is evidence that photoreceptor degeneration is closely related to vasculopathy in the diseased retina [8], [13], [14], [35]. We observed that RCS rats developed progressive capillary dropout and both intra- and sub-retinal neovascularization as they aged. Our observations are consistent with the concept that vascular atrophy observed in patients and animals with retinal degeneration are secondary to decreased metabolic demand as the photoreceptors die [35], [36]. Photoreceptors have the highest metabolic rate of any cell in the body [37]. Previous studies have shown reduced intraretinal oxygen distribution during photoreceptor degeneration in RCS rats, which reflects the gradual loss of oxygen metabolism of the degenerating photoreceptors [38], [39]. We observed progressive development of retinal gliosis and subretinal neovascularisation during photoreceptor degeneration in RCS rats. In the normal retina, retina glia including astrocytes, Müller cells and microglia, provide direct and indirect support to retinal neurons by upregulation of trophic factors and antioxidants [40], [41], [42]. As retinal glia are involved in the formation and regulation of blood-retinal barrier [31], [40], [42], glial dysfunction would inevitably affect the retinal vasculature [26], [43]. A recent study reported that aberrant vessel formation in RCS rats was accompanied by upregulation of angiogenic factors such as vascular endothelial growth factor, somatotropin release-inhibiting factor, angiopoietin-2 and their receptors [13]. These studies indicate that photoreceptor degeneration can have secondary effects on retinal vessels although the precise cellular and molecular mechanisms remain unclear.

Recent studies indicate that the biological effects of EPO extend beyond its traditional effects on regulation of erythropoiesis [15], [44], [45]. EPO has been reported to function in a paracrine manner to protect tissues during ischemic, toxic, and traumatic insults [46], [47], [48]. EPO is reported to inhibit cell apoptosis [20], [46], [49] and prevent ischemia-related damage in the brain [50], heart [51] and retina [18], [20], [52], [53]. Treatment with EPO or an EPO-derived peptide attenuated neuroglial and vascular degeneration without exacerbating pathological neovascularization in the retina [18], [20], [22], [52], [53], [54]. Neuronal damage and vasculopathy are often inter-dependent in retinal diseases. We chose to start EPO treatment at 14 weeks of age in RCS rats, a time point when only one or two rows of photoreceptors remain while subretinal abnormal vessels are just starting to develop, in order to minimize an effect of EPO on the vasculature that was mediated through it protective effect on photoreceptors. As expected, EPO inhibited photoreceptor apoptosis but this effect did not increase the thickness of outer nuclear layer since it was given after the photoreceptors had almost completely degenerated [10], [12]. Analysis of retinal vascular changes at that time indicated that systemic administration of EPO had prevented retinal capillary dropout and inhibited the development of focal vascular lesions. We then found that EPO inhibited retinal gliosis, as evidenced by the reduced expression of GFAP and attenuated intrusion of Muller cell processes in the subretinal space. The inhibitory effects of EPO on photoreceptor apoptosis and retinal gliosis we observed in this study are consistent with a previous study showing that EPO protected photoreceptors [21], [22] and inhibited the increased GFAP immunoreactivity [22] in Muller cells in rd mice. EPO treatment also increased the number of ramified microglial infiltration in the subretinal space and influenced differential expression of p75^NTR^ and pro-NT3. Taken together, our data suggest that the trophic effects of EPO treatment on photoreceptors and the retinal vasculature may involve multiple mechanisms including regulation of retinal glia and microglia as well as the p75^NTR^-pro-NT3 pathway signaling.

There is increasing evidence that bone marrow-derived progenitor cells can differentiate into functionally mature glia [35], [55], [56], microglia [20] and endothelial cells [57], [58] for vascular rescue. Recent studies reported that EPO increases the number and function of bone marrow derived endothelial progenitor cells in cardiovascular diseases [59], [60], [61], [62]. We found that EPO increased the numbers of red and white blood cells in peripheral blood. EPO treatment increased the number of CD34^+^ cells in the bone marrow and peripheral blood. Using a combination of CD34 with VEGF-R2 for flow cytometry, we found that EPO treatment enhanced the mobilization of CD34^+^/VEGF-R2^+^ cells from the bone marrow to the peripheral blood, which was reflected by a reduced number of CD34^+^/VEGF-R2^+^ cells in the bone marrow and an increased number of this phenotype of cells in the circulation.

We anticipated that the mobilized CD34^+^/VEGF-R2^+^ cells might incorporate into damaged retinal vessels of RCS rats in an attempt to repair them. Double label staining of retinal wholemounts showed EPO treatment significantly increased the number of CD34^+^ cells in the retina of RCS rats. However, most CD34^+^ cells were found in the superficial retina where they were scattered randomly. We noted that many CD34^+^ cells showed microglia-like morphology but they did not appear to preferentially localise around damaged retinal vessels. Immunofluorescence studies showed that EPO promoted the infiltration of ramified microglia into the subretinal space and choroid. The increased infiltration of microglia into EPO-treated retinas most likely originated from the mobilized bone marrow cells. Chen et al. [20] observed a similar phenomenon in a model of oxygen-induced retinopathy after EPO treatment. Previous studies have reported that resident microglia originate from the bone marrow and that CD34^+^ bone marrow progenitor cells can differentiate into perivascular and ramified microglia [63], [64]. Ritter et al. [65] reported that bone marrow-derived progenitor cells can migrate to avascular regions of the retina, differentiate into microglia and facilitate normalization of the vasculature in an animal model of oxygen-induced retinopathy. Taken together, our data combined with previous studies suggest that EPO can increase the number of ramified microglia and contribute to rescue of retinal vasculature in diseased conditions. Future research is warranted to study the beneficial and deleterious functions of different classes of microglia [66] for the maintenance of retinal vasculature and neuronal health.

The timing and dosing of EPO if it is to be used to treat retinal vasculopathy need to be carefully considered. This is particularly important for ischemic retinal conditions, such as the later stages of retinopathy of prematurity and retinal vein occlusion. There is in increasing evidence that treatment of ischemic retinas with EPO stimulates pathological neovascularization [20], [67], [68], [69]. Both EPO and VEGF play essential roles in the maintenance of retinal vasculature and photoreceptor health [70], [71], [72] but overexpression of these growth factors in ischemic retinas can be destructive [27], [67]. In our study, intraperitoneal injections of recombinant EPO in 14 week old RCS rats, twice a week for 4 weeks, increased VEGF expression by 38% but this did not accelerate the progression of vascular lesions.

In summary, we have shown here that systemic EPO treatment prevented retinal capillary dropout and focal vascular lesions in RCS rats. EPO modulated retinal gliosis and increased the infiltration of ramified microglia into the retina. EPO inhibited photoreceptor apoptosis, influenced differential expression of a number of molecules associated with retinal neuronal and vascular health and stimulated the production and mobilization of bone marrow derived cells. Our results warrant further investigation of EPO to treat neuronal and vascular pathology in retinal disease.
